# Book Reviews

**Published:** 1904-01

**Authors:** 


### BOOK REVIEWS.

---

PROGRESSIVE MEDICINE. Lea Bros. & Co., New York and Philadelphia, December, 1903.

This volume of the Progressive Medicine Series contains articles on the following subjects: Diseases of the Digestive Tract and Allied Organs, Liver Pancreas and Peritoneum, by John C. Hemmeter, M.D.; Anesthetics, Fractures, Dislocations, Amputations, Surgery of the Extremities and Orthopedics, by Joseph C. Bloodgood; Genito-Urinary Diseases, by W. T. Belfield; Diseases of the Kidneys, by John Rose Bradford; Physiology, by Albert P. Brubaker; Hygiene, by Chas. Harrington; Practical Therapeutic Referendum, by H. R. M. Landis.

The volume is well indexed and well printed, and the subjects treated are brought up to date of publication.

---

AT the meeting of the Society of Medical Jurisprudence, held on the 12th of November at the Academy of Medicine, a paper was read by Justice Julius M. Mayer of the Court of Special Sessions on "Criminal Procedure against the Unlawful Practice of Medicine," of which the *Medical Record* gives the following synopsis: "The worst agency in New York to-day," said the writer, "that helps the man who sells either real or pretended abortion medicine is the newspapers, for they make it possible to ensnare the unwary, the superstitious, and the fearful. I suggest that in the new school of journalism in Columbia there be a chair of advertising, and let it be taught to the young men of the newspaper profession that the first duty of a great newspaper is to censor its medical advertising. If the decent newspapers will assist in the gradual uplifting of public opinion concerning the men and women who engage in these disreputable and criminal occupations, it will be only a matter of a short time until they are driven out of business."

